# Predictors of working days lost due to sickness absence and disability pension

**DOI:** 10.1007/s00420-020-01630-6

**Published:** 2021-01-12

**Authors:** Rahman Shiri, Aapo Hiilamo, Ossi Rahkonen, Suzan J. W. Robroek, Olli Pietiläinen, Tea Lallukka

**Affiliations:** 1 Finnish Institute of Occupational Health, Työterveyslaitos, P.O. Box 18, 00032 Helsinki, Finland; 2 Department of Public Health, University of Helsinki, Helsinki, Finland; 3 Department of Public Health, Erasmus Medical Center Rotterdam, Rotterdam, The Netherlands

**Keywords:** Chronic disease, Healthy lifestyle, Life expectancy, Occupations, Sick leave, Workload, Work engagement

## Abstract

**Objective:**

To identify social and health-related predictors of the number of days lost due to sickness absence (SA) and disability pension (DP) among initially 55-year-old public-sector workers.

**Methods:**

The data from the Finnish Helsinki Health Study included participants aged 55 years at the baseline (in 2000–2002, *N* = 1630, 81% women), and were enriched with register-based information on SA and DP. The cumulative number of calendar days lost due to SA ≥ 1 day or DP between ages 55 and 65 was calculated. Negative binomial regression model was used to identify the predictors of days lost.

**Results:**

The average calendar days lost was 316 days (about 220 working days) during a 10-year follow-up, and 44% were due to SA and 56% due to DP. Smoking [incidence rate ratio (IRR) = 1.19, 95% CI 1.01–1.40 for past and IRR = 1.30, CI 1.07–1.58 for current], binge drinking (IRR = 1.22, CI 1.02–1.46), lifting or pulling/pushing heavy loads (IRR = 1.35, CI 1.10–1.65), awkward working positions (IRR = 1.24, CI 1.01–1.53), long-standing illness limiting work or daily activities (IRR = 2.32, CI 1.93–2.79), common mental disorder (IRR = 1.52, CI 1.30–1.79), and multisite pain (IRR = 1.50, CI 1.23–1.84) increased the number of days lost, while high level of education (IRR = 0.66, CI 0.52–0.82) and moderate level of leisure-time physical activity (IRR = 0.80, CI 0.67–0.94) reduced the number of days lost.

**Conclusions:**

Modifiable lifestyle risk factors, workload factors, common mental disorder, and multisite pain substantially increase the number of days lost. However, the findings of this study could be generalized to female workers in the public sector. Future research should also consider shorter SA spells in estimating working years lost and working life expectancy.

**Supplementary Information:**

The online version contains supplementary material available at 10.1007/s00420-020-01630-6.

## Introduction

Musculoskeletal and mental disorders are the most common causes of disability pension (DP) (Canivet et al. 2013; Kaila-Kangas et al. 2014; Knudsen et al. 2012). In 2019, mental and behavioral disorders surpassed musculoskeletal diseases as the number one cause of DP in Finland (Finnish Centre for Pensions 2020). Moreover, mental disorders can account for the higher number of working years lost due to DP than musculoskeletal disorders, because individuals retired due to mental disorders are generally younger than those retired due to musculoskeletal disorders (Knudsen et al. 2012). Earlier register-based studies have identified the following predictors of DP: a low level of education (Ahola et al. 2011; Bruusgaard et al. 2010), excess body mass (Robroek et al. 2013), smoking (Korhonen et al. 2015), low level of leisure-time physical activity (Fimland et al. 2015), problem drinking (Skogen et al. 2012), multisite pain (Haukka et al. 2015; Kamaleri et al. 2009), common mental disorders (Ahola et al. 2011), physical illnesses (Ahola et al. 2011), exposure to physical workload factors (Emberland et al. 2017; Järvholm et al. 2014; Kjellberg et al. 2016), and job strain (Ahola et al. 2011).

Despite the growing number of studies on DP and working life expectancy, a few studies have focused on working years lost due to short and long sickness absence (SA) and DP days combined. Most of the previous studies (Dudel et al. 2018; Dudel and Myrskylä 2017; Leinonen et al. 2018) focused only on working years lost due to DP. This approach underestimates working years lost and overestimates working life expectancy. This is because a notable part of absence from work is due to SA prior to the grant of part-time or permanent exit from paid employment. For example, in Finland, DP is typically granted only after it is preceded by SA of 1 year. Therefore, to more precisely estimate the duration of working years lost, both SA and DP days need to be considered. Furthermore, little is known about the length and predictors of working years lost due to SA and DP combined. While register data provide accurate and complete information on working years lost, information on predictors and confounding factors is largely unavailable from registers. To date, very few studies determined the predictors of combined SA and DP (Ervasti et al. 2017; Halonen et al. 2017; Lund et al. 2001). Furthermore, these studies did not include SA of less than 10 days, while most SA spells are short.

The aim of the present study was to estimate the number of working days lost due to SA and DP, and to identify social and health-related predictors of working days lost.

## Methods

### Population

**Fig. 1:**
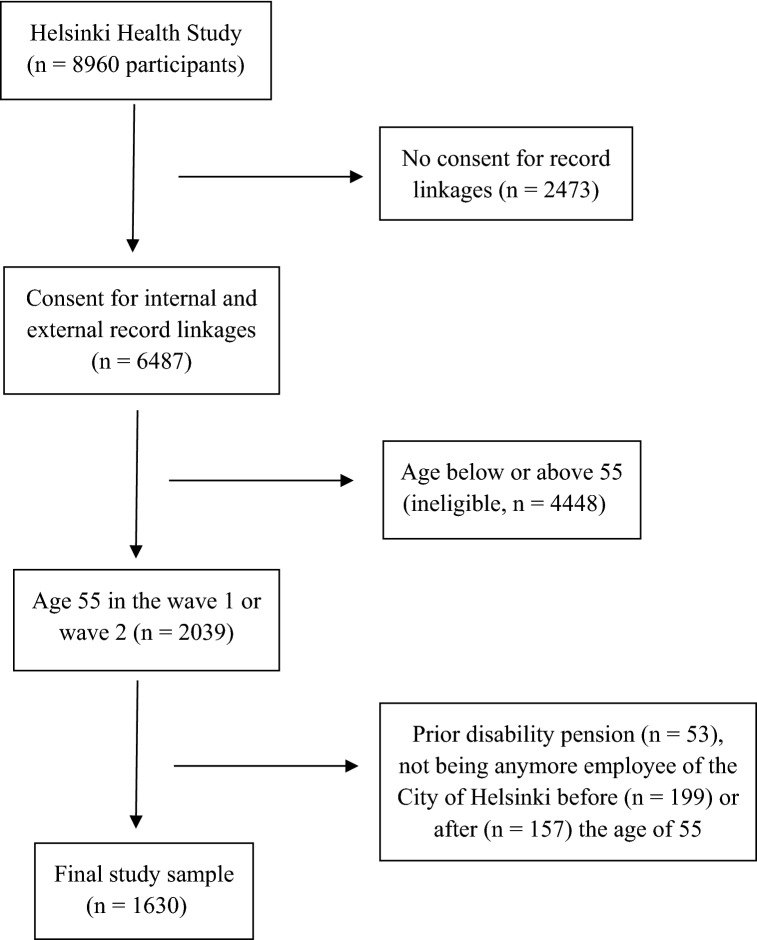
Flowchart of the study population

The population of this study was derived from the prospective Finnish Helsinki Health Study. The study is described in more detail elsewhere (Lahelma et al. 2013). The study recruited the employees aged 40–60 years of the City of Helsinki, the largest single employer in Finland. In the present analysis, we limited the study population to the participants aged 55 years at the baseline survey conducted during the years of 2000–2002 (response 73% for women and 61% for men, Laaksonen et al. 2008) or those aged 55 years at the first follow-up survey conducted in 2007 (Fig. 1). We included participants who provided informed consent for register linkages and remained employed by the same employer. The age of 55 years was selected to follow-up people for their working years lost before their statutory retirement age. Additionally, a large number of early exits take place after the age of 55. The final study population consisted of 1630 participants, of whom 81% were women, which reflects the female-dominated role of public-sector work in Finland. The ethics committees of the health authorities of the City of Helsinki, and the Department of Public Health, University of Helsinki approved the study.

### Number of days lost

Information on all sickness absence days was obtained from the personnel register of the City of Helsinki. The register comprises all self-certified short and medically certified longer SA days. The number of calendar days lost due to SA was calculated by the cumulative number of SA days between ages 55 and 65. Information on disability pension was obtained from the Finnish Centre for Pensions’ register. The number of calendar days lost due to DP was calculated from the start and end date of each DP award. Temporary DP has a fixed end date, while in continuing DP, the end date usually refers to the date on which DP is transferred to legal old age retirement. In Finland, disability pension can also be awarded as partial retirement, which requires part-time work. For the partial DP, we used half weights (i.e., a year on a partial retirement equals to 0.5 years lost).

### Independent variables

Baseline characteristics included gender, education (1. elementary school, 2. middle school, 3. vocational school or equivalent, 4. college degree, and 5. university degree), occupational class (1. managers and professionals [e.g., managers, administrators, teachers, and doctors], 2. semi-professionals [e.g., nurses, technicians, and foremen], 3. routine non-manual employees [e.g., clerical workers and healthcare assistants], and 4. manual workers [e.g., transport workers, cleaning workers, and canteen workers]) (Aittomäki 2008; Leinonen et al. 2011), smoking status (never, past, and current), binge drinking (6 units or more at the time in the past 30 days), body weight and height, leisure-time physical activity, work-related physical and psychosocial factors, self-reported long-standing illness, number of pain sites, and common mental disorders.

Body mass index (BMI) was calculated using self-reported weight and height and grouped into three levels: normal (BMI < 24.9 kg/m^2^), overweight (BMI 25.0–29.9 kg/m^2^), and obesity (BMI ≥ 30 kg/m^2^). Only seven participants had BMI < 18.5 kg/m^2^ and we included them in normal weight category. Excluding these seven participants from the analyses did not change the results. Information on the participants’ average weekly hours of leisure-time physical activity was gathered using four questions on: (1) walking, (2) brisk walking, (3) jogging, and (4) running. The number of hours per week for each activity ranged from 0 to 4 h. A metabolic equivalent (MET) index was calculated for each participant by multiplying the MET values of each activity intensity by the time spent on them, and summing them up (Ainsworth et al. 2011; Lallukka et al. 2016). The intensity of leisure-time physical activity was classified into three groups: low (< 14 MET-hours per week), moderate (≥ 14 MET-hours of moderate intensity activities with no vigorous activities), and vigorous activity (≥ 14 MET-hours, including some vigorous activity such as jogging or running). For the assessment of physical workload factors (8 items), working with a computer and mouse (2 items), and environmental factors (8 items), the 18-item inventory developed at the Finnish Institute of Occupational Health (Piirainen et al. 2003) was used. The workload factors included (1) heavy lifting, or pulling or pushing heavy loads; (2) back rotations; (3) awkward working positions; (4) repetitive movements; (5) vibration; (6) standing; (7); walking; and (8) sitting. Five items of Karasek’s Job Content Questionnaire (Karasek et al. 1998) were used to assess job demands and seven items to assess job control. Demand and control scales were dichotomized at the median and job strain was defined as reporting high job demands coupled with low job control.

Common mental disorder was assessed using the General Health Questionnaire (GHQ-12). We used a cut-point 3 or higher scores to define presence of a mental health problem (Goldberg et al. 1997). Data on the presence of acute/subacute (less than 3 months) or chronic (longer than 3 months) pain in the head/face, neck/shoulders, lower back, upper limbs, lower limbs, abdominal area, and any other location were collected by the self-administered questionnaires. We classified the number of pain sites into three groups (none, one, and two or more), and defined multisite pain as pain in two or more sites. Long-standing illness was grouped into illness limiting and not limiting work or daily activities.

### Statistical analysis

Missing data ranged from missing information on occupation for six participants to missing one or more items of leisure-time physical activity for 92 participants (5.6%). Fourteen percent of the participants had missing data on one or more independent variables. We imputed missing data using the method of multiple imputation by chained equations (van Buuren et al. 1999) and created 10 datasets. As a sensitivity analysis, we also ran complete case analysis for both genders combined. Spearman's rank correlation coefficient was used to estimate the correlation between two categorical variables and the variance inflation factor (VIF) was used to assess multicollinearity between independent variables. We first reported the observed average number of days lost by the baseline characteristics and then identified the predictors of days lost using negative binomial regression model. The model estimates rate as the number of days lost (a count) per unit time. Observation time for total number of days lost was calculated by the time between the age of 55 and statutory pension, death, or the age of 65. After accounting for the effects of the predictors, the variance of the number of days lost was larger than the mean, and the dispersion statistic was above one. Therefore, for this overdispersed count data, negative binomial regression was a better model than Poisson regression.

First, gender-specific or gender-adjusted incidence rate ratios (IRR) were estimated (base model) and then adjusted IRRs (full model) were estimated for both genders combined and for women due to a high number of women in the sample. The full model adjusted for gender, education, occupational class, smoking, body mass index, leisure-time physical activity, binge drinking, heavy lifting, back rotations, awkward working positions, long-standing illness, common mental disorder, and number of pain sites. For men, because of small sample size, the non-significant characteristics were removed from the full model one at a time until all characteristics were associated with the number of days lost with *P* value ≤ 0.2. The predicted number of days lost were calculated by user-written *mimrgns* command in Stata (StataCorp, College Station, TX, USA). The predicted number of days lost was calculated from the fitted full model, while keeping all other covariates at their means (using “atmeans” option) (Williams 2012).

## Results

Of the study participants, 19% were current smokers, 37% were overweight, and 19% obese at baseline (Table 1). Sixty-eight percent were moderately or vigorously active during leisure-time, 33% were exposed to heavy lifting, or pulling or pushing heavy loads, 43% were exposed to back rotations, and 50% to awkward working positions at baseline. Twenty-five percent had long-standing illness limiting their work or daily activities, 25% had multisite pain (pain in two or more body locations), and 26% reported a common mental disorder at baseline. Education was correlated with occupation (Spearman's rank correlation coefficient = 0.72), and back rotations was correlated with awkward working positions (Spearman's rank correlation coefficient = 0.69). The magnitude of correlation coefficient between other variables was less than 0.55. There was no indication of multicollinearity and the variance inflation factor was less than 2.3 for all independent variables.

**Table 1:** Gender-specific and both genders combined average number of days lost due to sickness absence or disability pension, and gender-specific or gender-adjusted incidence rate ratio (IRR) of number of days lost according to background characteristics at baseline

Characteristic	Both genders (*N*=1630)				Men (*N*=303)			Women (*N*=1327)		
	% of sample	Average number of days lost	IRR	95% CI	Average number of days lost	IRR	95% CI	Average number of days lost	IRR	95% CI
Overall	100	316			276			326		
Gender										
Women	81.4	326	1							
Men	18.6	276	0.92	0.76–1.10						
Education										
Elementary or middle school	23.6	470	1		401	1		481	1	
Vocational school or equivalent	22.7	410	0.84	0.68–1.03	400	0.73	0.41–1.30	412	0.86	0.69–1.07
College or university degree	53.7	210	0.47	0.39–0.56	202	0.35	0.22–0.57	212	0.50	0.41–0.60
Occupational class										
Managers or professionals	32.5	188	1		192	1		185	1	
Semi-professionals	17.9	297	1.65	1.34–2.03	394	1.97	1.25–3.11	270	1.56	1.23–1.97
Routine non-manual workers	36.3	351	1.87	1.57–2.23	425	2.06	1.11–3.83	347	1.85	1.54–2.23
Manual workers	13.3	564	2.95	2.35–3.70	293	2.41	1.49–3.90	656	3.12	2.41–4.04
Smoking										
Never	56.9	277	1		272	1		278	1	
Past	24.0	326	1.21	1.02–1.44	241	1.09	0.73–1.62	363	1.25	1.03–1.52
Current	19.1	421	1.43	1.19–1.73	348	1.33	0.82–2.17	439	1.45	1.18–1.78
Body mass index										
Normal	44.3	260	1		227	1		267	1	
Overweight	37.2	327	1.33	1.13–1.57	294	1.53	1.03–2.27	336	1.28	1.08–1.53
Obesity	18.5	430	1.62	1.33–1.97	333	1.36	0.82–2.27	452	1.67	1.35–2.06
Leisure-time physical activity										
Low	32.2	361	1		294	1		376	1	
Moderate	45.5	290	0.79	0.66–0.95	323	0.96	0.56–1.64	285	0.76	0.64–0.91
Vigorous	22.3	306	0.84	0.68–1.04	212	0.62	0.36–1.05	344	0.93	0.74–1.17
Binge drinking (once a month or more)										
No	79.9	301	1		262	1		307	1	
Yes	20.1	378	1.36	1.13–1.63	294	1.26	0.87–1.82	434	1.40	1.13–1.74
Heavy lifting, or pulling or pushing heavy loads										
No	67.1	225	1		238	1		221	1	
Yes	32.9	502	2.23	1.91–2.60	497	2.13	1.29–3.53	503	2.25	1.91–2.63
Back rotations										
No	57.0	227	1		252	1		219	1	
Yes	43.0	435	1.96	1.69–2.26	352	1.97	1.29–2.98	444	1.96	1.67–2.28
Awkward working positions										
No	50.0	222	1		278	1		202	1	
Yes	50.0	410	2.00	1.71–2.32	269	1.36	0.91–2.02	427	2.13	1.82–2.50
Job strain										
No	74.4	293	1		235	1		308	1	
Yes	25.6	384	1.34	1.14–1.59	454	2.00	1.26–3.17	373	1.24	1.04–1.48
Long-standing illness										
No	60.6	204	1		185	1		210	1	
Yes, not limiting work or daily activities	14.3	231	1.13	0.92–1.39	248	1.47	0.86–2.50	227	1.07	0.85–1.33
Yes, limiting work or daily activities	25.1	635	3.23	2.73–3.82	598	4.02	2.58–6.26	642	3.09	2.58–3.69
Common mental disorder										
No	74.1	250	1		205	1		260	1	
Yes	25.9	508	1.99	1.69–2.33	496	2.29	1.52–3.47	511	1.93	1.62–2.29
Number of pain sites										
None	52.6	197	1		205	1		195	1	
One	22.7	316	1.70	1.40–2.05	219	1.32	0.78–2.22	336	1.78	1.45–2.19
Two or more	24.7	571	2.94	2.44–3.54	668	3.43	2.04–5.76	559	2.89	2.38–3.50

During a 10-year follow-up period, average calendar days lost due to SA or disability pension was 316 (SD 654) days (Table 1), and the median was 77 days. Overall, 44% of days lost were due to SA and 56% were due to disability pension (Supplemental Table S1).

### Gender-adjusted model

The number of days lost did not differ between men and women (Table 1). The number of days lost in the participants with college or university degree was half of that of those with elementary or middle school education. Compared with managers or professionals, the number of days lost was higher in other occupational groups, particularly manual workers. Past and current smoking, overweight and obesity, binge drinking, heavy lifting, back rotations, awkward working positions, job strain, long-standing illness limiting work or daily activities, common mental disorder, and number of pain sites increased the number of days lost (Table 1). Leisure-time physical activity, particularly moderate level, reduced the number of days lost. Job control was associated with lower rate of days lost (IRR = 0.63, CI 0.56–0.71 for 1-unit increase), whereas job demands were not associated with the number of days lost ((IRR = 1.03, CI 0.93–1.14). In gender-specific analyses, the findings in women were similar to those of both gender-combined. In men, smoking, obesity, leisure-time physical activity, binge drinking, and awkward working positions were not statistically significantly associated with the number of days lost.

### Full multivariable model

**Fig. 2:**
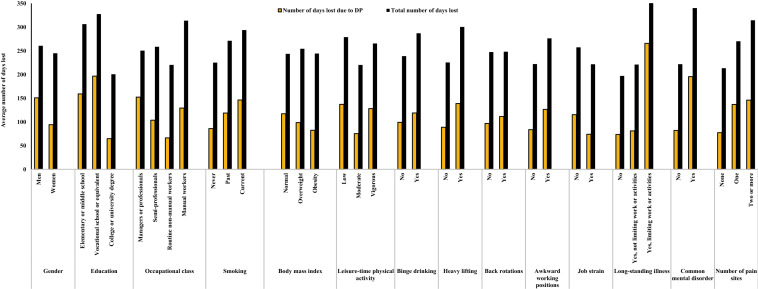
The predicted total number of days lost due to sickness absence and disability pension (DP) and the number of days lost due to only disability pension by background characteristics at baseline

Figure 2 presents the predicted number of days lost due to both SA and DP as well as the number of days lost due to only DP by background characteristics. Gender, occupational class, overweight and obesity, vigorous leisure-time physical activity, back rotations, and long-standing illness not limiting work or daily activities were not associated with the number of days lost in the model adjusted for all covariates (Table 2 and Fig. 1). Past and current smoking, binge drinking, heavy lifting, awkward working positions, long-standing illness limiting work or daily activities, common mental disorder, and number of pain sites increased the number of days lost, while high level of education and moderate level of leisure-time physical activity reduced the number of days lost. The number of days lost was also slightly lower in participants with job strain. Job demands and job control were not associated with the number of days lost. In subgroup analysis, job strain was associated with lower number of days lost among managers, professionals or semi-professionals, but not among routine non-manual workers or manual workers. In women, the associations were similar to those of both gender-combined, except that manual workers had significantly higher number of days lost compared with managers or professionals (Table 2). In men, current smoking, overweight, back rotations, long-standing illness limiting work or daily activities, and multisite pain increased the number of days lost (Table S2).

**Table 2:** Full model incidence rate ratio (IRR) of number of days lost due to sickness absence or disability pension according to background characteristics in both genders combined (*n *= 1630) and in women (*n* = 1327)

Characteristic	Both genders		Women	
	IRR	95% CI	IRR	95% CI
Gender, men vs. women	1.06	0.87–1.28		
Education (ref: elementary or middle school)				
Vocational school or equivalent	1.06	0.87–1.30	1.05	0.86–1.30
College or university degree	0.66	0.52–0.82	0.71	0.55–0.91
Occupational class (ref: managers or professionals)				
Semi-professionals	1.03	0.83–1.26	1.00	0.79–1.26
Routine non-manual workers	0.88	0.70–1.10	0.94	0.73–1.21
Manual workers	1.25	0.93–1.68	1.56	1.11–2.18
Smoking (ref: never)				
Past	1.19	1.01–1.40	1.25	1.04–1.50
Current	1.30	1.07–1.58	1.24	1.01–1.54
Body mass index (ref: normal)				
Overweight	1.06	0.91–1.24	1.03	0.87–1.21
Obesity	1.01	0.82–1.23	1.16	0.93–1.46
Leisure-time physical activity (ref: low)				
Moderate	0.80	0.67–0.94	0.81	0.69–0.96
Vigorous	0.98	0.79–1.23	1.16	0.93–1.46
Binge drinking (once a month or more vs. no)	1.22	1.02–1.46	1.32	1.07–1.64
Heavy lifting, or pulling or pushing heavy loads	1.35	1.10–1.65	1.31	1.06–1.61
Back rotations	0.99	0.82–1.21	0.91	0.74–1.12
Awkward working positions	1.24	1.01–1.53	1.45	1.16–1.81
Job strain	0.85	0.72–0.997	0.82	0.69–0.98
Long-standing illness (ref: no)				
Yes, not limiting work or daily activities	1.10	0.90–1.35	1.05	0.85–1.30
Yes, limiting work or daily activities	2.32	1.93–2.79	2.21	1.81–2.69
Common mental disorder	1.52	1.30–1.79	1.51	1.27–1.79
Number of pain sites (ref: none)				
One	1.29	1.07–1.55	1.47	1.20–1.79
Two or more	1.50	1.23–1.84	1.53	1.24–1.89

Table 3 shows the predicted number of days lost based on the full model according to the participants’ baseline characteristics. Long-standing illness limiting work or daily activities increased the number of days lost by 261 days (95% CI 191–332), common mental disorder by 118 days (95% CI 68–169), and multisite pain by 101 days (95% CI 47–155). The increase in the number of days lost for smoking, binge drinking, heavy lifting, and awkward working positions ranged between 46 and 75 days. On the other hand, moderate level of leisure-time physical activity reduced the number of days lost by 59 days (95% CI 15–102).

**Table 3:** The predicted number of days lost due to sickness absence or disability pension by sociodemographic, lifestyle, and occupational factors and medical conditions

Characteristic	*N* (days)	95% CI	*P* value
Education			
Vocational school or equivalent	21	− 42, 84	0.51
College or university degree	− 106	− 166, − 45	0.001
Smoking			
Past	46	4, 89	0.033
Current	69	17, 121	0.010
Leisure-time physical activity			
Moderate	− 59	− 102, − 15	0.008
Vigorous	− 13	− 68, 41	0.626
Binge drinking (once a month or more)	48	− 1, 98	0.057
Heavy lifting, or pulling or pushing heavy loads	75	24, 126	0.004
Awkward working positions	54	4, 105	0.036
Long-standing illness			
Not limiting work or daily activities	24	− 22, 69	0.306
Limiting work or daily activities	261	191, 332	< 0.001
Common mental disorder	118	68, 169	< 0.001
Number of pain sites			
One	57	3, 111	0.039
Two or more	101	47, 155	< 0.001

## Discussion

The present study found that 55 year old public-sector workers lose, on average, about 316 calendar days or about 220 working days because of SA and DP by the age of 65. Smoking, binge drinking, heavy lifting, awkward working positions, long-standing illness limiting work or daily activities, common mental disorder, and multisite pain increase the rate of working days lost, while high level of education and moderate level of physical activity during leisure time reduce the rate. However, the results of the current study mainly refer to female workers in the public sector, and generalization to other groups is not warranted or should be done with caution.

Most of the earlier studies on working years lost have not included SA spells in their estimations. They have not been able to consider short-term SA. The findings of the present study suggest that ignoring SA periods underestimates working years lost due to work disability. For example, in Finland, for granting a disability pension, long SA period is required before the award. In the current study, even though workers with adverse health conditions retired earlier and their risk of SA was lower; however, they spent a longer time in SA.

In line with an earlier study (Laaksonen et al. 2018), working years lost due to SA and DP differed between educational groups. Earlier studies found that workers in lower educational groups are at higher risk of SA/DP combined (Ervasti et al. 2017; Halonen et al. 2017) and lose more working years due to DP of all diagnostic categories (Laaksonen et al. 2018). The largest difference was seen for DP due to musculoskeletal disorders (Laaksonen et al. 2018). Between 2005 and 2014, working years lost due to DP decreased in all educational groups. However, educational differences in DP narrowed for somatic diseases, and increased for mental disorders (Laaksonen et al. 2018). In line with earlier studies (Ervasti et al. 2017; Halonen et al. 2017), manual workers were at a greater risk of work disability than managers or professionals. Moreover, previous studies found that very high physically demanding work, and exposure to lifting or carrying heavy loads increase the risk of SA/DP combined (Sundstrup et al. 2018). In the current study, the IRR for heavy lifting, or pulling or pushing heavy loads increased from 1.35 (CI 1.10–1.65) to 1.54 (CI 1.26–1.88) after removing education and occupation from the full model. The estimates did not change for other predictors.

Previous population-based prospective cohort studies showed the detrimental effect of multisite pain on DP (Haukka et al. 2015; Kamaleri et al. 2009; Øverland et al. 2012). Multisite pain increases not only the risk of DP due to musculoskeletal disorders, but also the risk of DP due to mental disorders (Haukka et al. 2015; Øverland et al. 2012). The risk of DP (Haukka et al. 2015; Kamaleri et al. 2009; Øverland et al. 2012), particularly DP due to musculoskeletal disorders (Haukka et al. 2015; Øverland et al. 2012), increases with increasing the number of pain sites. Common mental disorders also increase the number of working days lost due to SA (Knudsen et al. 2013) and DP (Kaila-Kangas et al. 2014; Mykletun et al. 2006). A meta-analytic review found that mental ill-health increases the risk of DP by 1.8-fold (van Rijn et al. 2014). Moreover, common mental disorders and musculoskeletal pain strengthen each other’s effect on DP (Dorner et al. 2016). Despite the substantial body of evidence, the previous research did not estimate how many working days lost due to SA and DP are attributed to multisite pain and common mental disorders. The current study adds to the existing body of knowledge that in midlife workers aged 55 years, common mental disorder increases the number of calendar days lost due to SA and DP combined by 118 days and multisite pain by 101 days by the age of 65. The results, however, could be generalized to female workers in the public sector.

Earlier, the Finnish Helsinki Health Study showed that obesity increases the risk of SA due to musculoskeletal and mental disorders (Svärd et al. 2020). In the current analysis, overweight (IRR = 1.20, 95% CI 1.02–1.40) and obesity (IRR = 1.23, 95% CI 1.00–1.50) were associated with the number of days lost after adjustment for gender, occupational class, smoking, leisure-time physical activity, binge drinking, heavy lifting, back rotations, awkward working positions, and common mental disorder. However, the associations disappeared after further adjustment for education, long-standing illness, and a number of pain sites. All three variables, particularly the number of pain sites, attenuated the association between BMI and the number of days lost. It seems that excess body mass increases the number of days lost through causing multisite pain and long-standing illness limiting work or daily activities.

Interventions for improving working conditions or reducing multisite pain and symptoms of common mental disorders or promoting physical activity during leisure time can reduce the number of working days lost and improve working life expectancy. Interventions targeting more than one domain are recommended (Cullen et al. 2018), although improving working conditions can also have an impact on the pain and symptoms of common mental disorders.

### Strengths and limitations

This study utilized data from an occupational cohort with good response rates, highly reliable and complete register-based work disability measures (Laaksonen et al. 2008; Roos et al. 2013), and a long follow-up time to estimate working days lost among midlife employees until the desired retirement age. Moreover, we were able to distinguish between SA and DP days, and combine all SA spells of different lengths for a more comprehensive picture of days lost as compared to previous studies that have mostly missed particularly a large number of short SA spells. We also had a large set of different potentially modifiable work and health-related predictors of working days lost, to identify potential groups for intervention and prevention. Based on earlier studies, we included the determinants of DP and SA in the full models; however, it cannot be ruled out that we have not missed some potential predictors of working days lost. Finally, including a homogenous group of people of the same age working for the same employer helps produce more reliable results. Despite these strengths, the study had some limitations. First, the assessments of weight and height, leisure-time physical activity, smoking, drinking, occupational physical workload factors, common mental disorder, and long-standing illness were self-reported; however, differences in their predictive power for SA have been shown to be small in this cohort (Korpela et al. 2013). Second, we did not have information on sickness absence for the employees who changed their employer during the follow-up period. However, only a small number of the participants aged 55 or older changed their employer. Third, the number of men was small, and thus, the results for men should be interpreted with caution.

## Conclusions

Smoking, binge drinking, occupational workload factors, long-standing illness, common mental disorder, and multisite pain substantially increase the risk of working years lost. On the other hand, a higher educational level and moderate level of leisure-time physical activity reduce the risk. Future research should also consider shorter SA spells in calculating working years lost and working life expectancy.

## Supplementary Information

Below is the link to the electronic supplementary material.

Supplementary file1 (DOCX 155 KB)
