# Supplementary material for: Predictors of working days lost due to sickness absence and disability pension
Source: Int Arch Occup Environ Health. 2021 Jan 12;94(5):843–54. doi: 10.1007/s00420-020-01630-6 (PMC8238732; doi:10.1007/s00420-020-01630-6)
Supplement: Supplementary file 1 — Supplementary file1 (DOCX 155 KB) [file 420_2020_1630_MOESM1_ESM.pdf]

Supplementary material

Predictors of working days lost due to sickness absence and disability pension

Rahman Shiri,[1] Aapo Hiilamo,[1] Ossi Rahkonen,[2] Suzan JW Robroek,[3] Olli Pietiläinen,[2] Tea Lallukka[1,2]

[1] Finnish Institute of Occupational Health, Helsinki, Finland

[2] Department of Public Health, University of Helsinki, Helsinki, Finland

[3] Department of Public Health, Erasmus Medical Center Rotterdam, Rotterdam, Netherlands

Table S1: Complete case analysis of average number of days lost due to sickness absence and disability pension and gender-adjusted incidence rate ratio (IRR) of total number of days lost due to sickness absence or disability pension according to background characteristics at baseline

Characteristic	N	%	Average number of days lost		IRR	95% CI
			Sickness absence	Disability pension		
Overall	1630	100	139	179		
Gender						
Women	1327	81.4	147	180	1	
Men	303	18.6	105	171	0.92	0.77-1.10
Education						
Elementary or middle school	381	23.5	188	286	1	
Vocational school or equivalent	367	22.7	174	242	0.84	0.68-1.03
College or university degree	871	53.8	103	107	0.47	0.39-0.55
Occupational class						
Managers or professionals	528	32.5	86	102	1	
Semi-professionals	290	17.9	130	170	1.66	1.35-2.03
Routine non-manual workers	589	36.3	165	186	1.88	1.58-2.24
Manual workers	217	13.3	209	360	2.96	2.36-3.71
Smoking						
Never	921	56.9	125	151	1	
Past	388	24.0	145	183	1.22	1.02-1.45
Current	309	19.1	173	250	1.44	1.19-1.73
Body mass index						
Normal	716	44.3	117	143	1	
Overweight	602	37.2	143	184	1.33	1.13-1.55
Obesity	299	18.5	182	250	1.63	1.34-1.98
Leisure-time physical activity						
Low	498	32.4	150	215	1	
Moderate	701	45.6	142	154	0.79	0.67-0.93
Vigorous	339	22.0	123	189	0.83	0.68-1.01
Binge drinking (once a month or more)						
No	1273	79.9	135	168	1	
Yes	321	20.1	154	227	1.34	1.12-1.61
Heavy lifting, or pulling or pushing heavy loads						
No	1079	67.3	111	112	1	
Yes	523	32.7	193	305	2.22	1.91-2.58
Back rotations						
No	915	57.0	109	114	1	
Yes	689	43.0	177	260	1.99	1.72-2.30
Awkward working positions						

Characteristic	N	%	Average number of days lost		IRR	95% CI
			Sickness absence	Disability pension		
No	804	50.2	106	112	1	
Yes	798	49.8	170	239	2.03	1.75-2.34
Job strain						
No	1201	74.5	133	161	1	
Yes	412	25.5	157	225	1.35	1.15-1.59
Long-standing illness						
No	954	60.8	104	97	1	
Yes, not limiting work or daily activities	226	14.4	130	97	1.15	0.94-1.41
Yes, limiting work or daily activities	389	24.8	220	423	3.33	2.83-3.93
Common mental disorder						
No	1204	74.2	126	123	1	
Yes	418	25.8	175	331	1.98	1.68-2.32
Number of pain sites						
None	799	53.1	100	89	1	
One	341	22.7	142	165	1.70	1.42-2.03
Two or more	364	24.2	209	350	2.98	2.50-3.55

Table S2: Full model incidence rate ratio (IRR) of days lost due to sickness absence or disability pension according to background characteristics at baseline in men

Characteristic	IRR	95% CI
Smoking (ref: never)		
Past	1.13	0.75-1.70
Current	2.16	1.32-3.54
Body mass index (ref: normal)		
Overweight	1.66	1.12-2.46
Obesity	0.81	0.47-1.39
Binge drinking (once a month or more vs. no)	1.32	0.91-1.91
Back rotations	2.06	1.08-3.92
Awkward working positions	0.55	0.30-1.01
Long-standing illness (ref: no)		
Yes, not limiting work or daily activities	1.38	0.80-2.38
Yes, limiting work or daily activities	2.67	1.61-4.41
Common mental disorder	1.48	0.95-2.30
Number of pain sites (ref: none)		
One	0.80	0.38-1.65
Two or more	2.60	1.45-4.65
